# Is Yield Increase Sufficient to Achieve Food Security in China?

**DOI:** 10.1371/journal.pone.0116430

**Published:** 2015-02-13

**Authors:** Xing Wei, Zhao Zhang, Peijun Shi, Pin Wang, Yi Chen, Xiao Song, Fulu Tao

**Affiliations:** 1 State Key Laboratory of Earth Surface Processes and Resources Ecology / Key Laboratory of Environmental Change and Natural Disaster, MOE / Academy of Disaster Reduction and Emergency Management, Beijing Normal University, Beijing, 100875, China; 2 Institute of Geographical Sciences and Natural Resources Research, Chinese Academy of Sciences, Beijing, 100101, China; Virginia Tech, UNITED STATES

## Abstract

Increasing demand for food, driven by unprecedented population growth and increasing consumption, will keep challenging food security in China. Although cereal yields have substantially improved during the last three decades, whether it will keep thriving to meet the increasing demand is not known yet. Thus, an integrated analysis on the trends of crop yield and cultivated area is essential to better understand current state of food security in China, especially on county scale. So far, yield stagnation has extensively dominated the main cereal-growing areas across China. Rice yield is facing the most severe stagnation that 53.9% counties tracked in the study have stagnated significantly, followed by maize (42.4%) and wheat (41.9%). As another important element for production sustainability, but often neglected is the planted area patterns. It has been further demonstrated that the loss in productive arable land for rice and wheat have dramatically increased the pressure on achieving food security. Not only a great deal of the planted areas have stagnated since 1980, but also collapsed. 48.4% and 54.4% of rice- and wheat-growing counties have lost their cropland areas to varying degrees. Besides, 27.6% and 35.8% of them have retrograded below the level of the 1980s. The combined influence (both loss in yield and area) has determined the crop sustainable production in China to be pessimistic for rice and wheat, and consequently no surprise to find that more than half of counties rank a lower level of production sustainability. Therefore, given the potential yield increase in wheat and maize, as well as substantial area loss of rice and wheat, the possible targeted adaptation measures for both yield and cropping area is required at county scale. Moreover, policies on food trade, alongside advocation of low calorie diets, reducing food loss and waste can help to enhance food security.

## Introduction

It has been widely accepted that advanced technology and management practice can benefit crop yield. Hybrid cultivars, together with precise agricultural managements, have significantly improved cereal yields for rice, wheat and maize [1–5]. However, increasing population has led to unprecedented food demand challenge and the expanding demand will be continued in the future [6]. Will yield increment from technology and management be sufficient for closing the gap between food supply and demand? And is this increment liable to be continuous or even thriving for the following years?

Unfortunately, the answer is obvious as we have already been challenged by the extensive yield stagnation around the world [7]. Climatic and technological ceilings are both key boundaries to further improve crop yield at field [8,9]. Specifically, natural endowment determines yield capacity thresholds and alongside global warming has caused yield reduction in several main crops [10–15]. Besides, excessive fertilizer applications have brought varying degrees of soil acidification and ecological degradation to the current arable land resources [16–19]. Moreover, increasing agronomic input has resulted in diminishing returns of land among crops thus yields have stagnated extensively [7,20]. Therefore, more and more emphasis has been put on promoting crop yield by closing yield gap between actual field yield and potential yield [7,21]. Yield-based analysis has provided a perspective of yield change patterns over the world given different resolutions of regional and sub-regional data [21,22]. Basically, these studies on yield promotion have an important premise that global crop area is stable or increasing [20,22].

However, land use changes are disparate around the world. Regions or countries which have lost large amount of arable areas may barely maintain food self-sufficiency in the foreseeable future. That is exactly the case for China and other countries which are experiencing a rapid economic development. Besides large proportion of cropland has encountered yield stagnation [7,9], arable area of main cereal crops has also shrunk during the last 30 years [23]. As the pivotal position of China in the world food market, food production and food security of China has always been a worldwide issue [6,16,24–26]. To achieve food security in China, consequently, both needs for yield improvement on existing croplands and area protection on productive farmland [7,20] are prerequisites. Therefore, yield-based analysis is only meaningful to understand the real situation of food security under an assumption of the stable arable land available. Previous studies only focusing on yield potential and yield change won’t be sufficient to evaluate comprehensively the food security in China, or even in the world. Hence, the sustainable development of crop production in China will, to a great extent, rely on expanding our knowledge on spatio-temporal variability in both crop yield and planted area. Although a few studies have noted the importance of closing yield gaps and also the substantial existence of decreasing cropland [27], no such studies have carried out specifying China till to date to our knowledge. Filling this vicinity presents great importance in achieving food security for China, and even worldwide. Besides, county is a basic and important administrative unit for agricultural management and implement of agriculture policies in China. Particularly in the case of China, counties even adjacent to each other may have highly diverse agronomic environments and types of agriculture development [9]. Being fully aware of food security is a multi-dimension problem including food availability, food access, food utilization and food stability [28], we mainly focus our analysis on food availability in China. For a great populous country like China, increasing domestic food production plays a fundamental role in improving food availability, thus achieving food security. Taking deep look into the trends of crop yield and cultivated area on the county scale is therefore of great significance and necessity for understanding the latest situation of food availability and food security in China.

Based on the county-level data of crop yield and planted area for rice, wheat and maize from 1980 to 2008, therefore, the main objectivities are: 1) to identify the dominant change patterns of both key factors (yield and planted areas) for three main cereals; 2) to comprehensively assess production sustainability (the ability to provide cereal production continuously) for each crop in each county; 3) and to seek a potential road for food security in China. This work is the first to assess synthetically food security at a county scale. The potential findings would give a remarkably improved understanding of cereal production sustainability across China, and would provide a paradigm for other countries in the world.

## Materials and Methods

### Data sources and pretreatment

Yield data of rice, wheat and maize and the related planted area from 1980 to 2008 were collected from Agricultural Yearbook of each county (published annually by the China Agriculture Press in Beijing) [29], China statistical yearbook (1980–2008) [30], and the publicly available records from public network [31]. The data quality was further controlled by removing the outliers that fell out the range of mean value ±2 times the standard deviation. Moreover, the counties were selected only if the records of yield and the corresponding planted area of each crop have spanned more than 15 years [22]. Totally, 1632, 1962 and 2061 counties for rice, wheat and maize yield analysis and 1155, 1389 and 1028 counties for rice, wheat and maize area analysis have met these pre-requirements, and been chosen respectively in the study. The intersection data set of yield and area for rice, wheat and maize contains 1088, 1273 and 962 counties respectively.

### Methods to identify the temporal trends of both crop yield and area

We generally followed Ray’s approach [7] to determine the temporal patterns of both crop yield and area, except for a normalization pre-process to maximize the quality and compatibility of data. During the data pre-process, firstly, the baseline value was set as the first record in a certain time series data for yield and planted area, respectively. A normalized new series of yield or area is then obtained by dividing the baseline value for each county. It is reported that the pre-process can present more actually the change type of each time series [32].

Then for each county, we performed four representations of yield-time and area-time functions, including an intercept only model (1a) and (1b), a linear model (2a) and (2b), a quadratic model (3a) and (3b) and a cubic model (4a) and (4b) which have shown in the following functions.

$$\{\begin{array}{ll} Yield=k_{y} & \left(1a\right)\\ Yield=a_{y}t+k_{y} & \left(2a\right)\\ Yield=a_{y}t^{2}+b_{y}t+k_{y} & \left(3a\right)\\ Yield=a_{y}t^{3}+b_{y}t^{2}+c_{y}t+k_{y} & \left(4a\right) \end{array}\{\begin{array}{ll} Area=k_{r} & \left(1b\right)\\ Area=a_{r}t+k_{r} & \left(2b\right)\\ Area=a_{r}t^{2}+b_{r}t+k_{r} & \left(3b\right)\\ Area=a_{r}t^{3}+b_{r}t^{2}+c_{r}t+k_{r} & \left(4b\right) \end{array}$$

where *Yield* denotes yield in tons ha^-1^ year^-1^, *Area* denotes area in ha and *t* denotes year; *k* is the intercept of regression; *a*, *b*, and *c* are the coefficients of regression; subscript *y* and *r* denote yield-time and area-time relationship respectively.

We next calculated Akaike Information Criterion (AIC) [33] for each model to identify parsimoniously the best fit model for each yield-time and area-time relationships. The AIC function is given by:

$$AIC=n\log(\frac{SS}{n})+2\rho$$ (5)

where *SS* is residual sum of squares, *n* is the sample size, and *ρ* is the number of parameters. The minimum AIC value corresponds to the best representation of the trend for a given province.

In addition, through incorporating with *F*-test, a test on overall goodness, we tested the chosen models with the lowest AIC value against the null hypothesis of the constant model (*p*<0.05). According to the chosen model parameters, yields and areas were classified into four categories: never improved, increasing, stagnated and collapsed.

The detailed information on such classification is: 1) The “never improved” type includes the selected model being an intercept only model and that has failed in *F*-test. 2) The “increasing” type includes the selected model being a linear model with a positive slope, a quadratic model with a positive sign of quadratic term or a negative sign of quadratic term but peaking beyond 2011, a cubic model with peaking beyond 2011. Some cases in which yield or area fluctuated significantly but increased in recent years are also classified into “increasing” category. 3) The “stagnated” type includes the selected model being a quadratic model with a negative sign of quadratic term and peaking between 1980 and 2011, a cubic model with peaking during 1980 and 2011. 4) The “collapsed” type includes the selected model being a linear model with a negative slope, a quadratic model with a negative sign of quadratic term and recent yield reaching below 1980s level. Some cases in which yield or area during 2006–2011 reached below the level of 1980–1985 are also classified into “collapsed” category.

### Methods to identify the food production sustainability

**Fig 1:**
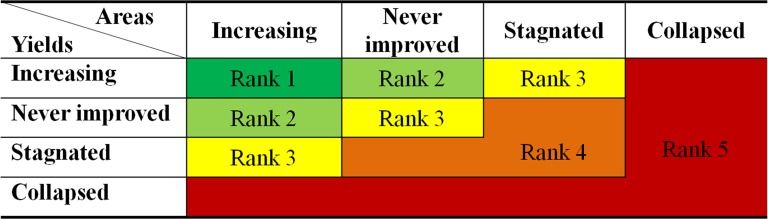
Identification matrix of food productivity sustainability ranks. Sustainability ranks based on yield-area trend relationships. Ranks diagonally increase from 1 to 5, suggesting decreasing sustainability of food productivity.

In the study, we are trying to figure out whether the food production is sustainable, that is to be produced continuously. Given the significant contribution of both yield and area to food production sustainability, herein we identify sustainability ranks by the two-dimensional matrix based on their relationships with time (Fig. 1). An increasing trend in crop yield or area implies the highest level of sustainability. Yield or area never improved in some provinces, as it doesn’t show a falling tendency, ranks the second level of sustainability in the four types. Trend that has stagnated is liable to continue shrink in yield or area, is less sustainable in our definition. The collapsed yield or area is identified as the least sustainable, where yield or area has retrogressed to the level of 1980s, suggesting a crumbling agronomic input or derelict agricultural land. The intersection data set mentioned in *Data sources and pretreatment section* is used here for sustainability analysis.

## Results

### Spatial distribution of yield trend type

**Fig 2:**
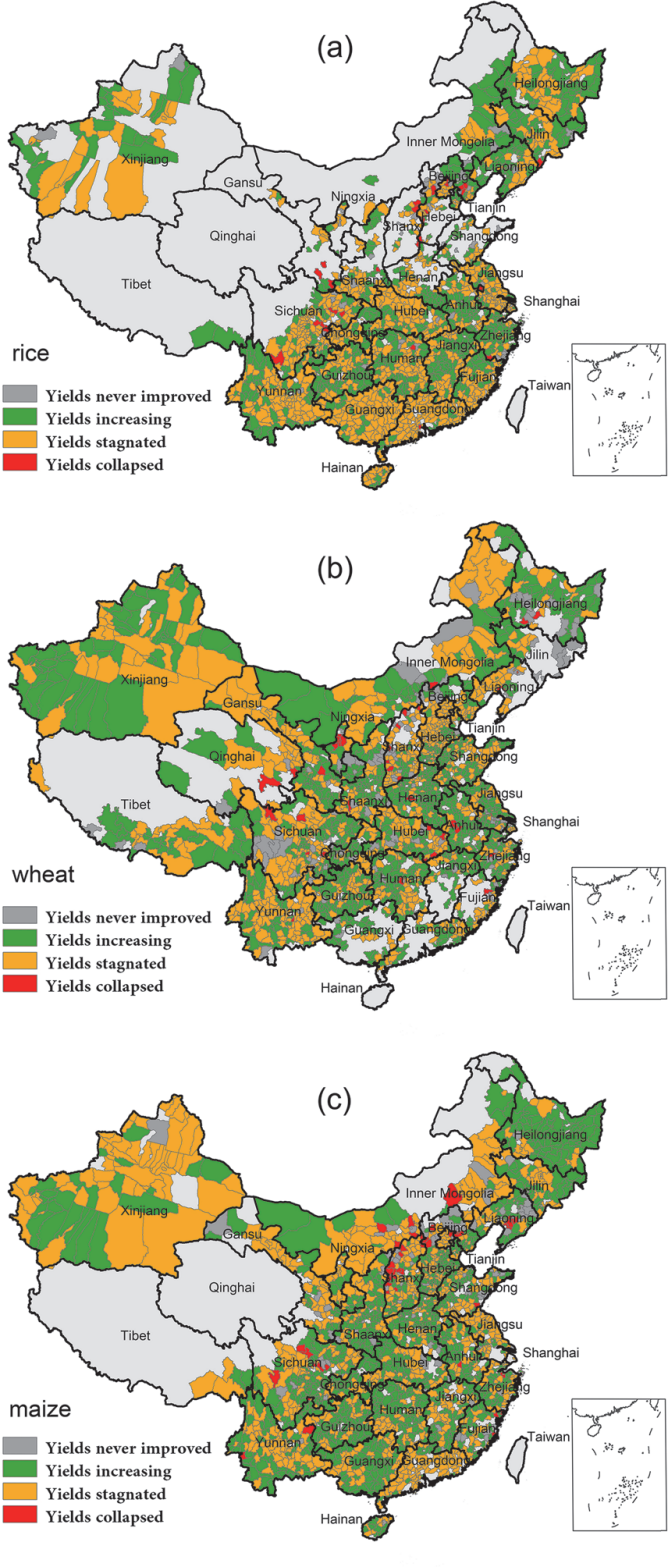
Pattern of the yield trends for main cereals. At each county where rice (a), wheat (b) and maize (c) crop yield were tracked, yield trends were divided into four types and color coded.

Our analysis shows that most of the mainland China has historically experienced yield increase during the last three decades for the three main cereal crops (Fig. 2).

The yield trend of rice is shown in Fig. 2A. Approximately 95.0% of the 1632 rice-growing counties we traced have experienced an improved yield (including both increasing and stagnated types) based on the 1980s level. Among these, however, 53.9% of them are witnessing yield stagnation wide spread across the studied region (Table 1). The southeast coastal and southern counties have suffered substantial yield stagnation, especially. Besides, yields of the central part of the northeast and northwest and central China also have stagnated. Only 3.3% of the rice-growing counties have yields that never improved, scatteredly located in some northern counties and along the border of the northwest (Table 1, Fig. 2A). Similarly, the proportion of yield collapse for rice is also very small (1.7%). These counties are sporadically distributed mainly in the southwest and the north.

**Table 1:** Types of trend patterns of yield and planted area and sustainability ranks of productivity.

	Type	Rice	Wheat	Maize
Yield	Never improved	54 (*3*.*31%*)	130(*6*.*63%*)	107(*5*.*19%*)
	Increasing	671(*41*.*12%*)	969(*49*.*39%*)	1034(*50*.*17%*)
	Stagnated	879(*53*.*86%*)	823(*41*.*95%*)	874(*42*.*41%*)
	Collapsed	28(*1*.*72%*)	40(*2*.*04%*)	46(*2*.*23%*)
	Sum	1632	1962	2061
Area	Never improved	175(*15*.*15%*)	116(*8*.*35%*)	80(*7*.*78%*)
	Increasing	102(*8*.*83%*)	21(*1*.*51%*)	374(*36*.*38%*)
	Stagnated	559(*48*.*40%*)	755(*54*.*36%*)	557(*54*.*18%*)
	Collapsed	319(*27*.*62%*)	497(*35*.*78%*)	17(*1*.*65%*)
	Sum	1155	1389	1028
Sustainability	Rank 1	44(*4*.*04%*)	5(*0*.*39%*)	171(*17*.*78%*)
	Rank 2	76(*6*.*99%*)	43(*3*.*38%*)	56(*5*.*82%*)
	Rank 3	257(*23*.*62%*)	418(*32*.*84%*)	419(*43*.*56%*)
	Rank 4	396(*36*.*40%*)	354(*27*.*81%*)	276(*28*.*69%*)
	Rank 5	315(*28*.*95%*)	453(*35*.*59%*)	40(*4*.*16%*)
	Sum	1088	1273	962

The percentage is the ratio of number of counties relative to tracked counties.

As for wheat yield of the 1962 counties, which cover the vast majority of northern and central China, yield has also substantially increased during the last three decades (Fig. 2B). However, different form rice yield, proportion of yield that is still increasing (49.4%) is more than that of stagnation (42.0%), as shown in Table 1. The eastern and central parts of the studied region are dominated by the increasing trend while yield stagnation has been wide prevailing in the southwest, the western parts of the northeast and the central parts of the northwest. Proportions of yield that has collapsed or never improved are also relatively lower compared with the types mentioned above, that is 6.6% and 2.0% respectively (Table 1). Counties with a never improved trend are generally clustered in the eastern parts of the northeastern and the southwestern China. However, collapsed yield trend locates mainly in the central parts of China (Fig. 2B).

As compared with the two crops above, maize has the largest proportion of yield that belongs to the increasing type (50.2%) for 2061 counties, almost half of the counties we traced. Even so, yield stagnation is also wide spread in maize-growing counties, accounting for 42.4% of the studied areas (Table 1). Yield has prominently stagnated in the northwest, north and western parts of the northeast, alongside with the border of the south and southeast (Fig. 2C). The proportion of yield collapsed of maize (2.2%) rates the top first among the three crops, 29.7% and 9.3% higher than rice and wheat respectively. These counties are mainly assembled at the north and southwest.

### Spatial distribution of the trend of crop area planted

Generally, the temporal patterns of area are evidently different from those of the yield. Area patterns of rice and wheat are dominated by the stagnated and collapsed types. Besides, maize-growing area has also suffered significantly from area stagnation.

**Fig 3:**
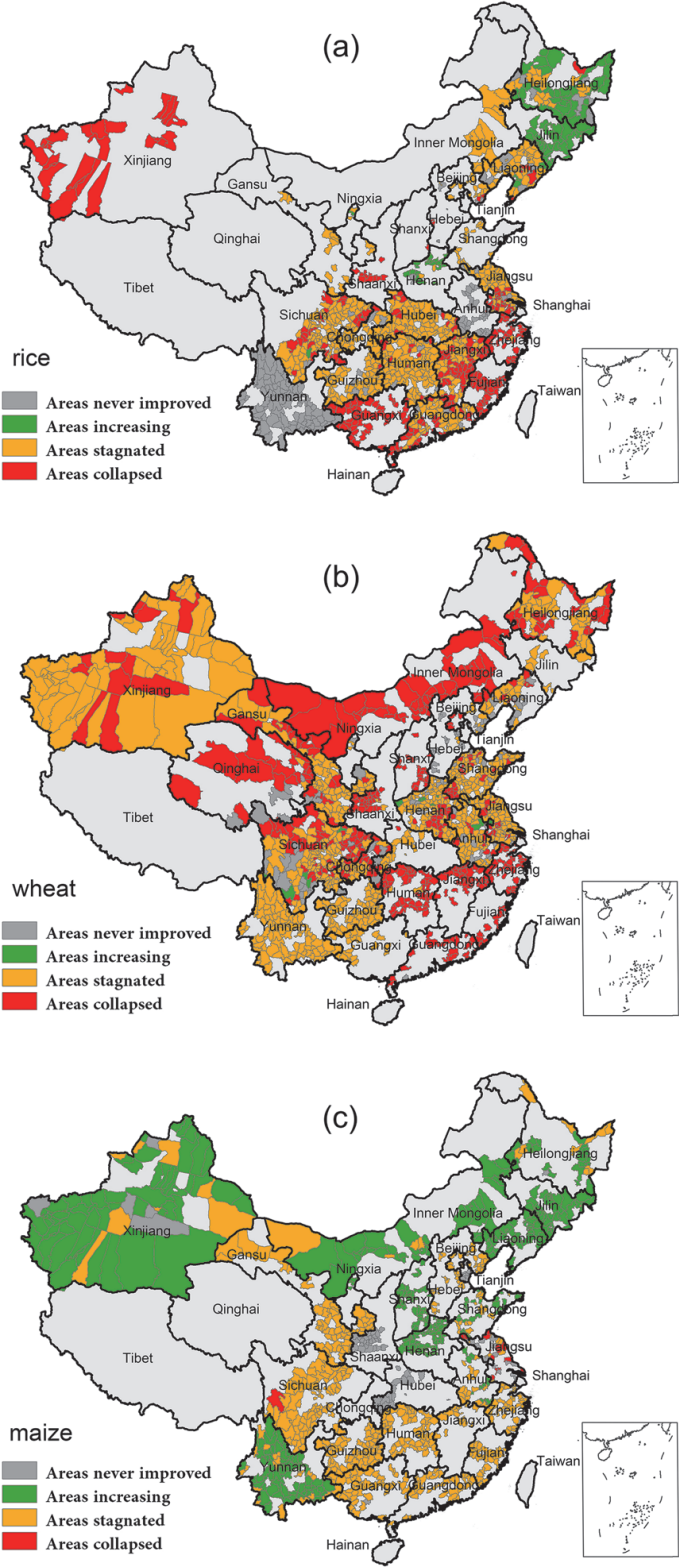
Pattern of the planted area trends for main cereals. At each county where rice (a), wheat (b) and maize (c) crop-growing area were tracked, area trends were divided into four types and employed the same color strategy.

Majority of the rice-growing counties, about 48.4% of the 1155 counties we traced, have stagnated in rice planted area (Table 1). Most of these counties distributed among the east, central part of the south and western part of the northeast (Fig. 3A). Increasing trend of area for planting rice only accounts for 8.8% of the whole studied region, mainly assembling at the Northeast China. On the contrary, area proportions of not improved and collapsed are relative larger compared with those of rice yield. 15.2% of the counties with never improved area are mostly distributed in the southwest and the east. And 27.6% counties with a collapsed area, which should be of great concern, lies along the southeast coast (Table 1, Fig. 3A).

Similarly but being more worrisome, the main areas for planting wheat have been swept by stagnation and collapse. 54.4% and 35.8% counties have been suffered by area stagnation and collapse, respectively, and the sum exceeds 90% of the whole counties (Table 1). Collapse is mostly distributed along the southeast coast and the north. Area stagnation mainly lies in the northwest, southwest and east (Fig. 3B).

As compared with those two crops above, the pattern of maize area is relatively less pessimistic. Although, more than half of the 1028 counties (54.2%) we traced have stagnated in area, there are still 36.4% of counties sharing an increasing trend of planted area for maize cultivation (Table 1). The southern, eastern and central China is the main contributor to the area stagnation. And the western, northern, northwestern and some parts of southwestern China are witnessing a substantial increasing pattern in area during the last three decades. Proportions for the never improved and collapsed areas are prominently lower than rice and wheat, only accounting for 7.8% and 1.7%, respectively, and mainly locating in the central part of China (Table 1, Fig. 3C).

### Spatial distribution of food production sustainability

Based on the definition of production sustainability, crop yield and planted area equal the same role contributing the sustainability rank. Thus, from our result of sustainability patterns, rice and wheat are of less sustainability due to the poor performance in area trends, while maize sustainability is better, attributing from both increment in yield and planted area.

**Fig 4:**
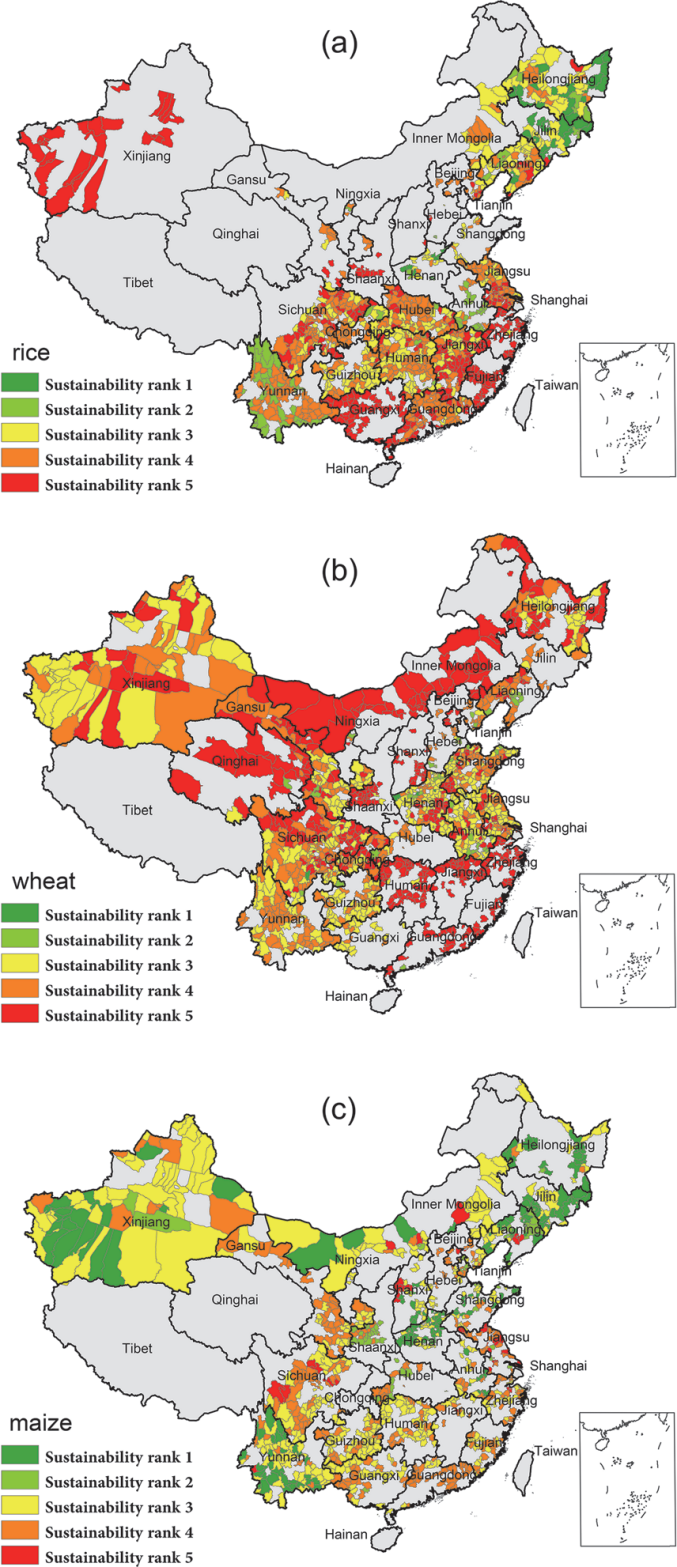
Pattern of the productivity sustainability for main cereals. Combining effects of both yield and area on production sustainability were color coded followed by the Tab.1 strategy.

Most of the 1088 counties for rice cultivation, intersected by both yield and area, ranks low at the 3^rd^, 4^th^ and 5^th^ level, with the proportion of 23.6, 36.4 and 29.0% respectively (Table 1). The least sustainable counties are located along the southeast coast, the northwest and some parts of the central China, followed by the counties of rank 4 in orange, mainly located in the southern and eastern parts. By contrast, rank 1 and 2 together accounts for 11.0%. East of the Northeastern China and Yunnan Province in the southwest rank the top first for sustainable rice productivity in China (Fig. 4A).

Sustainability of wheat productivity can be barely attainable according to our result. The proportion of the least level of sustainability (rank 5) has the largest share (35.6%) among all levels for wheat, and also compared with those of rice (29.0%) and maize (4.2%) (Table 1). Furthermore, the sum of lower sustainable counties (rank 3, 4 and 5) contribute to 96.2% of the whole 1273 counties. These counties are wide distributed across the studied region, especially in the north and southeast. However, counties with higher sustainability (rank 1 and 2) are only scattered among those with lower levels (Fig. 4B).

As for maize sustainability, compared with those two crops above, the situation is more promising to a certain extent. Share of rank 1 reach 17.8%, about 3 times and 40 times larger than those of rice and wheat. The majority (43.6%) of the total 962 counties rank the medium level (rank 3). And the least sustainable counties only accounts for 4.2% (Table 1). Moreover, the northeast and southwest areas are also more sustainable than other regions (Fig. 4C).

## Discussion

### Historical changes in food production from 1980 to 2008

**Fig 5:**
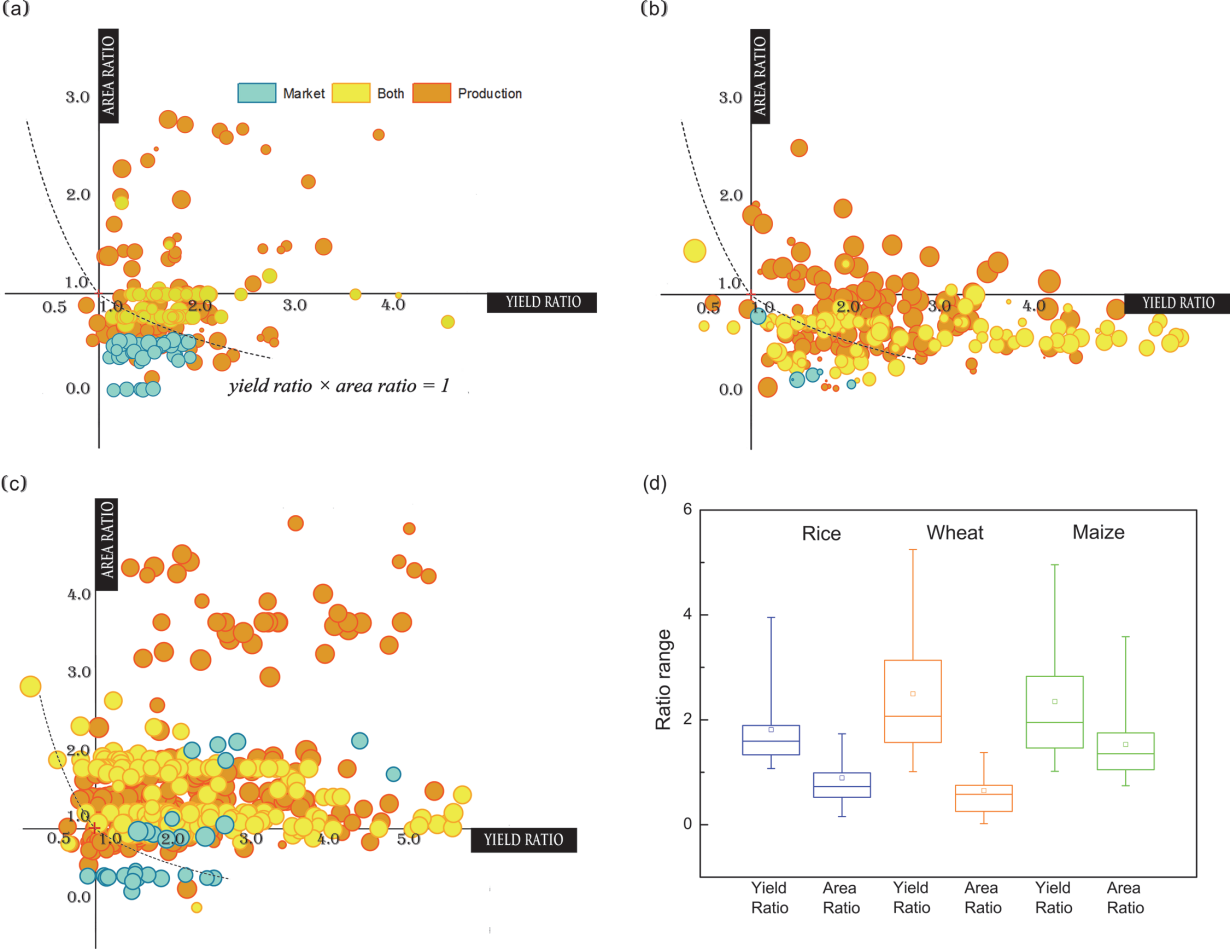
Static comparison in production between 1980 and 2008 of the main cereals. We compared the static change in yield, area and production for rice (a), wheat (b) and maize (c) undertaking different assignment of crop production and/or marketing, from 1980 to 2008. The horizontal and vertical axis shows changes in yield and area. Size of each circle shows the ratio of area in 2008 relative to 1980. Blue points denote counties assigned for marketing, while yellow points for both production and marketing, and orange for production. The ratio greater than 1 implies an increasing trend in yield or area. If the ratio is less than 1, yield or area has decreased from 1980 to 2008. Change in production is expressed by the product of yield ratio multiplying area ratio. Dots above the dotted line imply increasing production from 1980 to 2008. The statistical relationships of yield and area ratio among the three cereal crops are illustrated in figure (d).

In this study, we focus on the food availability, one aspect of food security and also the most basic and fundamental one. China has failed in self-sufficiency and become a net importer [23,34]. Thus, the importance of food availability has spoken for itself. And food production is one of the key elements of food availability. To detect the changes of food production from 1980 to 2008, we compare the static variation in between. Fig. 5 shows the comparison of yield and planted area for rice, wheat and maize between 1980 and 2008. The majority of counties for crop cultivation have witnessed yield increasing from 1980 to 2008. The rice yield ratio of 90% (5%-95%) counties ranges from 1.1 to 4.0. Comparatively speaking, the ratios range more largely for wheat and maize, with the interval of 1.0–5.3 and 1.0–5.0 respectively, considerably wider than the range of rice. Thus, wheat and maize yield may probably have more potential to improve than rice, based on the 1980’s level. This finding is consistent with the analysis on yield trends, in which the rice yield stagnation is the most severe because more than half counties have encountered a halted yield since 1980 (Table 1). Moreover, yield increase in more than three quarters of rice-growing counties barely doubled during 30-year cultivation (Fig. 5D), indicating that the rate of rice yield growth is comparatively lower. This may be ascribed to the closing yield gap of rice to meet the threshold value of yield potential [5,21]. Therefore, investments on the wheat and maize yields, also the low yield lands that have not been fully developed will be more promising to further improve cereal yield in the future.

Despite the existing trouble of yield stagnation, the situation of the arable land is more worrisome. Several previous studies have also stated that large tracts of cropland have been abandoned or replaced by constructions due to soil degradation and other political, social-economic or ecological factors [35–37]. The area ranges of three cereals are all smaller than those of yield, especially for rice and wheat (Fig. 5D). Approximately 80.1% and 88.5% of the rice- and wheat-growing counties have lost their arable areas. Such finding is substantiated by those of area trend analysis, in which the arable lands have been lost for the majority of rice- and wheat- growing counties, with the area ratio less than 1 and lying below the horizon axe (Fig. 3A, B, Fig. 5A, B). Most counties (78.9%) for maize cultivation, on the contrary, have experienced an extensive increase in cultivated area since 1980. One intriguing and also profound change is the transition between wheat and maize cultivation. Former wheat-growing counties collapsed their planted area to favor maize, especially in the northern and northeastern China (Fig. 3B, C). Limited water availability and increasing irrigation cost have stimulated this transition [38–40]. Maize is considered as a crop with higher water productivity/water use efficiency, suggesting less water requirement for irrigation while providing the same production of wheat [41,42]. Even so prominent the transition, area changes in cropping land and their influence on cereal production are still overlooked, compared with the previous studies overemphasizing yield assessment and estimation.

### Reasons for the spatial distribution of food production in China

From our analysis, it’s obvious that not only improving yield on the existing cereal lands, but also holding the productive cropland area is of great significance to achieve food security in China, particularly in the case of rice and wheat. For example, we make the first pre-assumption that the size of arable land that decreased in 2008 keeps the original level of 1980. Then the crop production will be 1.2, 2.0 and 1.0 times higher than the actual production of rice, wheat and maize in 2008, respectively. Such result suggests a profound and significant role of keeping the existing cropland on cereal production. However, such assumption above implies the expansion of cropland, which is not supportive to our degraded ecosystem or the real in nowadays China. So the second pre-assumption we made is that we keep all cropping area in 2008 the same size as 1980, that is, the processed area size in 2008 is exact the size of 1980. Then the production will be 99.72, 195.94 and 61.41% of that in 1980, for rice, wheat and maize respectively. Totally, based on the historical envisage, it has been demonstrated that rice yield increase can almost compensate the loss of production result from expanding cropping area, while area loss has substantially decreased wheat production. More importantly, the remarkable prosperity in maize production should be ascribed to great sprawl cultivation in Northeast and Southwest China.

Distribution, another aspect of food availability beside production, has substantially changed during the study period. To see more clearly the situation of food distribution in China, agricultural function division is introduced for a further comparison. The agricultural function division dates back to 1994 when the State Council Notice on Deepening the Reform of the Structure of Grain Procurement and Marketing was released. And such structure reform lasted until the final decision on agricultural function division which was made in 2001. Based on the gap between production and marketing within a certain province, the government divided its provincial administrative units into three categories: market counties, production counties, and counties for both. According to this division, Beijing, Tianjin, Shanghai‚ and other 4 provinces became market of domestic food trading system. Heilongjiang, Jilin, Liaoning and altogether 13 provinces were assigned for food production for the whole nation. And the other 11 provinces were about to achieve balance between crop production and marketing [43]. Following this classification, unfortunately, it is indicated apparently that cropping area decreases in almost all market counties (colored in blue) for rice and wheat growth and partial of maize growth (Fig. 5A-C). Moreover, these counties with a decrease in cropping areas of rice and wheat might also have experienced the yield decrease, and both decreases can consequently resulting in dramatic reduction in cereal production (Fig. 5A-C). Production counties and the third counties (counties for both market and production) perform fairly on yield improvement while more cropping area increase has happened in production counties than the third ones since 1980. In summary, rice- and wheat-growing counties mainly face the severe reduction in cropping area while maize-growing counties improve their production strongly depending on expansion in acreage.

### The potential way for the sustainability development of food production

We had great prosperity in agro-development before, and crop yield has dramatically improved, like a fivefold increment in production, thanks to the technological, political, and management practices, even the climatic benefit [20,44–47]. The increasing trend, however, has halted or even collapsed in some counties according to the findings of the study. At present, we have two and only two ways to promote crop production, increasing yield on the existing arable land or farming more land on the planet that already heavily loaded [20,48]. However, due to the request of minimizing the expense of environmental quality, closing yield gap between the actual yield and the potential should be our first priority [5,21,22,49]. Technology such as development of high-yield cultivars through targeted plant breeding and also the high-yield management practices have brought profound yield increase that does result in low yield gaps in China [3,46,50,51]. Such low yield gaps have been indicated by rice in eastern, central and southern China, wheat in eastern China and maize in central and northeastern China [8,9,48]. Concurrently, despite current yield in some regions has reached the threshold of 80% of their yield potential [52], great yield gaps still exist in all of the three main cereal crops [8,48]. For further utilizing the favorable climatic and soil resources, more chemical, nutrient and water inputs will be in great need to close these gaps [44]. Therefore, the tradeoff between ecological degradation, driven by these intensive land management practices, and increasing demand for crop production will be of great concern within the safe operating space for humanity [53,54].

### The significant implications of the study for food security

In the study, we note that food security cannot be achieved merely base on the food availability. Although we concentrate on domestic food production, we are also acknowledging the importance of the other dimensions of food security (food access, food utilization and food stability). Through analyzing spatio-temporal patterns of both yield and planted area for Chinese main cereals, we find that our food production won’t be sustainable until the main drivers, yield and area, are growing under control. Such findings have substantiated strongly the mass stagnation in rice and wheat yield (Fig. 2A, B), while yield of maize has stagnated in a relatively moderate manner (Fig. 2C), which are comparable to Ray’s [7] result of yield on global scale and Zhang’s study which only focused on rice in China [9]. Besides, we construct the planted area patterns, as well as the yield analysis, to provide a better understanding of current food availability in China. Considering the significant role of county as a basic administrative unit during Chinese agricultural activities, we conducted the first comprehensive and systematic study on production sustainability at a county scale. Thus, our results are competent to answer the question—how will we achieve food security in China, especially when the former savior of food production, yield increase, showing significant stagnation and decline.

Speaking of food availability, reducing food losses and waste can save around one quarter of the total food production globally, while the figure in China is about 20% [55,56]. The large quantity of food lost and wasted along the food supply chain has reduced the efficiency of resource utilization. Hence, reduction in food losses and waste could lead to a significant increase in food availability around the world, and for China as well. China feeds 21% of the world’s population with only 6% of world’s water resources and 9% of the world’s arable land. A higher efficiency of food supply chain will substantially reduce pressure on production. And on the side of food demand, China is in great need of more food to feed. One prominent change is the diet change following with the drastic development of the economic reform. The shift not only results in increase of calorie intake, but also higher proportion of cereal in diets [57]. And the cereal demand may keep increasing for human consumption and for livestock feed due to increase in animal production consumption [34,58]. Thus, following a simple diet and limited animal production consumption would also decrease pressure on cereal production [59]. In addition to domestic cereal production, world trade plays an important role in distribution of global food resources [34,60]. A strong economic is required to feed the country depending on food import [34]. Thus, considering the great population, no wonder domestic food production is so important to take a deep insight into as we have behaved the study. Besides, well functioned import policies based on precisely examined extent of dependence on other countries may be essential for securing food availability [60].

Moreover, other factors such as food access (related with affordability, allocation and preference), food utilization (related with nutritional value, social value and food safety) and food stability (related with food availability, access, utilization over time) should been involved for a more comprehensive assessment on food security. Generally, price mechanisms and policies, economic growth, stability and government support would help achieving food security in China [61]. And as an alternative, next generation biorefineries could effectively mitigate the stress of agriculture and perhaps address the trilemma among food, biofules and environment [62,63].

Although our analysis is limited to picture details of food security, it has managed to capture the most important element, food availability, in China. Furthermore, the domestic food production sustainability is challenged by stagnated yield increment and unexpected arable land shrink. Several measures among dimensions of food security are suggested. Together with all these actions, food security can probably be guaranteed in a sustainable way.

## Conclusions

In this study, to answer the question whether current crop yield increase will be sufficient to achieve food security in China, we explored the temporal patterns of yield and planted area, and assess the level of production sustainability for three main cereal crops at county scale. Results show that yield increase have slowed down and even halted among rice, wheat and maize. Thus, keeping high yield of rice and upgrading medium- and low-yield fields of wheat and maize may be of more rationality and feasibility to further increase cereal yields approaching their potential yield. Additionally, the original findings of area change pattern show stagnation and collapse are prevailing in most growing areas of rice and wheat, making their production sustainability under great threat. By contrast, maize production is both prominent for yield and area trends in comparison with rice and wheat, especially for area. Upgrading medium- and low-yielding fields, other than expanding maize-growing areas is more beneficial in the environmental way. Thus, this integrated trend analysis on cereal yield and planted area in China provides the prototype to better understand production sustainability elsewhere in the world.

Achieving food security needs long-term perspective, not only adequate food supply, but also considering nutritional content, individual preference and so on. The macroscopic strategies covering yield improvement, cropland conservation, modification in nutritional construction, and food trade are of substantial significance for sustainable development of agriculture to achieve food security in China.
