# Correction: Is Yield Increase Sufficient to Achieve Food Security in China?

**DOI:** 10.1371/journal.pone.0222167

**Published:** 2019-08-30

**Authors:** Xing Wei, Zhao Zhang, Peijun Shi, Pin Wang, Yi Chen, Xiao Song, Fulu Tao

S1 File is omitted from the article. It can be viewed below.

## Supporting information

S1 File Data access information. (DOCX)

## References

[1] Wei X, Zhang Z, Shi P, Wang P, Chen Y, Song X, et al (2015) Is Yield Increase Sufficient to Achieve Food Security in China? PLoS ONE 10(2): e0116430 10.1371/journal.pone.0116430 25680193 PMC4332688

