# Supplementary material for: Correction: Is Yield Increase Sufficient to Achieve Food Security in China?
Source: PLoS One. 2019 Aug 30;14(8):e0222167. doi: 10.1371/journal.pone.0222167 (PMC6716774; doi:10.1371/journal.pone.0222167)
Supplement: S1 File — (DOCX) [file pone.0222167.s001.docx]

**Supporting Information**

Yield data of rice, wheat and maize and the related planted area from 1980 to 2008 were collected from Agricultural Yearbook of each province or county published annually (<http://tongji.cnki.net/overseas/engnavi/navidefault.aspx>), and the publicly available records from public network. For example, some of the data are available from the <http://tongji.cnki.net/overseas/engnavi/navidefault.aspx> and the National Earth System Science Data Sharing Infrastructure (<http://www.geodata.cn/>) by searching the keywords such as ‘crop yield’ and ‘county’ in Chinese. The authors obtained these data from the National Earth System Science Data Sharing Infrastructure (<http://www.geodata.cn/>) and the Agricultural Information Institute, Chinese Academy of Agricultural Science (<http://aii.caas.cn/>) who collected the data. The data are owned by the National Earth System Science Data Sharing Infrastructure (<http://www.geodata.cn/>) and the Agricultural Information Institute, Chinese Academy of Agricultural Science (<http://aii.caas.cn/>).

We selected the yield data of rice, wheat and maize and the related planted area from 1980 to 2008 for this study. The data quality was further controlled by removing the outliers that fell out the range of mean value ±2 times the standard deviation. Moreover, the counties were selected only if the records of yield and the corresponding planted area of each crop have spanned more than 15 years. Totally, 1632, 1962 and 2061 counties for rice, wheat and maize yield analysis and 1155, 1389 and 1028 counties for rice, wheat and maize area analysis have met these pre-requirements, and been chosen respectively in the study. The intersection data set of yield and area for rice, wheat and maize contains 1088, 1273 and 962 counties, respectively.
